# A Mixed Kijima Model Using the Weibull-Based Generalized Renewal Processes

**DOI:** 10.1371/journal.pone.0133772

**Published:** 2015-07-21

**Authors:** Ricardo José Ferreira, Paulo Renato Alves Firmino, Cláudio Tadeu Cristino

**Affiliations:** 1 Department of Statistics and Informatics—Post-Graduate Program in Biometry and Applied Statistics, Federal Rural University of Pernambuco, Recife, Pernambuco, Brazil; 2 Federal University of Cariri, Juazeiro do Norte, Ceará, Brazil; Universidad de Valladolid, SPAIN

## Abstract

Generalized Renewal Processes are useful for approaching the rejuvenation of dynamical systems resulting from planned or unplanned interventions. We present new perspectives for the Generalized Renewal Processes in general and for the Weibull-based Generalized Renewal Processes in particular. Disregarding from literature, we present a mixed Generalized Renewal Processes approach involving Kijima Type I and II models, allowing one to infer the impact of distinct interventions on the performance of the system under study. The first and second theoretical moments of this model are introduced as well as its maximum likelihood estimation and random sampling approaches. In order to illustrate the usefulness of the proposed Weibull-based Generalized Renewal Processes model, some real data sets involving improving, stable, and deteriorating systems are used.

## Introduction

Generalized Renewal Process (GRP) is a well-known modelling approach to treat dynamical systems exposed to planned or unplanned interventions. This modelling is traditionally made by appending a rejuvenation parameter, say *q*, in the parameter set of a given probability distribution. This idea was firstly presented by [1] who introduced the concept of virtual age—a function that operates on the real age of the system via *q*. Thus, if *q* = 0 the GRP approaches a Renewal Processes (RP); otherwise, if *q* = 1 it represents a Non-Homogeneous Poisson Processes (NHPP). As, in the cases of deteriorating(improving) systems, the hazard function is directly(inversely) proportional to the age, the better the interventions the lesser(greater) the virtual age, always decreasing hazard characteristics underlying the system. Several examples in literature, such as [2–5], and [6] make use of this reasoning.

Authors like [2, 3], and [7] have resorted to the Weibull-based GRP (WGRP). In these cases, the parameters set of the WGRP is thus (*α*, *β*, *q*), representing, in this order, the scale, shape, and virtual age parameters. Though their unarguable establishment and increasing use through literature, GRP suffer from the lack of theoretical bases and flexibility in some circumstances. For instance, in their most widely known versions, the virtual age functions aim to measure the impact of each intervention on either the last time between interventions (Kijima Type I) or on the whole set of times between interventions (Kijima Type II); however, the literature does not present some kind of decision rule for choosing between these two structures in the light of a given data set or even an alternative involving a degrade between these two extremes of modelling for the impact of the interventions. Reflecting these scenarios [7] present some theoretical results only for Kijima Type I and other authors does not specify which of the Kijima models have used in their works, such as [3, 8, 9]. Further, some authors like [10] have stated that the closed-form solutions of some of GRP statistical metrics (*e*.*g*. the mean time between interventions) are not available.

In order to address these drawbacks, this paper presents a mixed GRP model where the virtual age is constructed by coupling Kijima Types I and II models. To date, real world dynamical systems frequently involve different classes of interventions, thus impacting on the recent (Kijima Type I), complete (Kijima Type II) or intermediate history of the system (proposed mixed model). From literature, [2] presents a model that capture the rejuvenation parameter for two classes of intervention—corrective and preventive maintenances. This coupling is performed here by appending the class label of each intervention *i*, say *y*_*i*_, and the respective coefficients, say *θ*_*y*_*i*__ and (1 − *θ*_*y*_*i*__), in a linear combination between the Kijima Types I and II models. Thus, when computing the virtual age, if *θ*_*y*_*i*__ = 1 then an intervention of class *y*_*i*_ only influence on the time since the last intervention (Kijima I) whilst if *θ*_*y*_*i*__ = 0 such influence is also on the current virtual age (Kijima II) and therefore on the complete history of the system maintenance. Otherwise, if *θ*_*y*_*i*__ ∈ (0,1) then the influence of the *i* ^*th*^ intervention intermediately impacts on the history of the system.

On the other hand, there is an important gap in literature concerning reliability databases. A review presented by [11] shows that just a minor part of researchers constructs a well structured reliability database with sufficient information to a robust analysis. Thus, it is rare to find informations detailing the nature of the interventions. Furthermore, a study involving Kijima models requires specific information regarding the type of the interventions, which has not been easily found in literature.

Also, [12] make a robust review about several authors that develop models based on GRP and Kijima development. Between the presented works, [13] present a generalization of Kijima virtual age models, but uses a dependent-time virtual age to compose their model. Thus, their model is an alternative to Kijima models, using the operational time to indicate the repair effectiveness. The idea of this paper is to measure the impact of different types of intervention by means of a mixed Kijima-based model.

Regarding WGRP, the math developed for the proposed model is presented analytically and numerically. The theoretical first and second moments as well as the maximum likelihood estimation problem and the inverse generation function are studied. Finally, the adjustment to some real data sets is presented and comparisons among five models—RP, NHPP, Kijima I, Kijima II, and the proposed approach (named Mixed Kijima model). These comparisons are based on the log-likelihood and the Mean Squared Error metrics.

The paper is structured as follows. Section 2 brings a brief presentation of WGRP and some new features. Section 3 brings new theoretical and numerical results to WGRP. Section 4 presents applications of the proposed model in real world cases whilst Section 5 brings some concluding remarks.

## The GRP modelling and some new features

In this section, we present known and new elements of GRP in general and WGRP in particular. In this way, some notations focused on the problem of fitting WGRP models to time series performance data sets involving the occurrence of events of interest in a given system are considered. Specifically, the *events of interest* will be considered as intended or unintended *interventions* on the condition of the system, and the focus will be on modelling the response of the system to these interventions in terms of the times between next interventions. Each intervention might be demanded by a single event from a set of possible (and eventually competing) ones (*e*.*g*. preventive and corrective actions) in such a way that the considered GRP model will incorporate the nature of such interventions in the modelling.

Without loss of generality, the word *time* will represent any unit measure over which the interventions are observed (*e*.*g*. meters, seconds, kilograms, cubic meters, and so on). Besides, the time during intervention is considered negligible, *i*.*e*. just point process are taken into account [14]. Finally, it is also considered that systematic increasing (decreasing) times between interventions characterize improvement (deterioration) of the system.

### The mixed virtual age

**Definition 1** *Let T_i_ be the time when the i^th^ intervention occurs (the actual cumulative time until i^th^ intervention) and let X_i_ be the time between the (i − 1)^th^ and the i^th^ interventions (X_0_ is a non-negative constant).*

From both Definition 1 and point processes foundations, we can see that $T_{i}=\sum_{j=1}^{i}X_{j}$ is the “real” age of the system when the i^*th*^ intervention occurs. A direct consequence is that *T*_0_ = *X*_0_. It has been usual to assume *X*_0_ = 0 in practice. It must also be highlighted that *X*_*i*_ (and therefore *T*_*i*_) can be characterized as random variables and thus subject to statistical modelling via GRP, once they can depend on the stochastic nature of the system condition. In turn, it is also reasonable to interpret the random vectors **T** = (*T*_1_, *T*_2_, …, *T*_*n*_) and **X** = (*X*_1_, *X*_2_, …, *X*_*n*_) as stochastic time series.

**Definition 2** *Let V_i_ be the virtual age of the system reflecting its restoration after i interventions. Thus V_i_ is a function of* ${\left\{X_{j}\right\}}_{j=1}^{i}$, *of the respective intervention types* ${\left\{Y_{j}\right\}}_{j=1}^{i}$, *and of an appending parameter, say q, V_i_ = v((X_1_, Y_1_), (X_2_, Y_2_), …, (X_i_, Y_i_) ∣ q).*

From Definition 2, proposed here, the GRP virtual age function can fit the performance data set of the system in terms of both the times between interventions **X** = (*X*_1_, *X*_2_, …, *X*_*n*_) and the respective nature of such interventions **Y** = (*Y*_1_, …, *Y*_*n*_) (*e*.*g*. whether planned or unplanned), besides the already known parameter *q*. To date, in the GRP literature, the vector **Y** is not taken into account.

Based on the observed time series, say ${\left\{x_{j},y_{j}\right\}}_{j=1}^{i-1}$, we have

$$v_{i-1}=v\left(\left(x_{1},y_{1}\right),\left(x_{2},y_{2}\right),...,\left(x_{i-1},y_{i-1}\right)\;|\;q\right).$$

In summary, it is suggested here that the level of restoration imposed to the system by each intervention might depends on the respective intervention type.

Besides **Y**, it is proposed here the following generalized Kijima-based virtual age model:

$$V_{i}=v\left(X_{i},Y_{i}\;|\;q,V_{i-1}\right)=\theta_{Y_{i}}\left(V_{i-1}+qX_{i}\right)+\left(1-\theta_{Y_{i}}\right)q\left(V_{i-1}+X_{i}\right),$$ (1)

where *θ*_*Y*_*i*__ ∈ [0, 1] and *q* ∈ ℝ. Thus, Eq (1) is a linear combination in such a way that *θ*_*Y*_*i*__ = 1 (*θ*_*Y*_*i*__ = 0) leads to the Kijima type I (Kijima type II) model. Therefore, considering *k* alternatives (intervention types) for *Y*_*j*_ one has *k* new parameters to GRP, say *θ* = (*θ*_1_,⋯, *θ*_*k*_) such that *θ*_*Y*_*i*__ ∈ *θ*, measuring the degrade between Kijima I and II models imposed to the system by each intervention type.

In Eq (1), for *θ*_*Y*_*i*__ = 1, the impact of the *i* ^*th*^ intervention only operates on *X*_*i*_, by *qX*_*i*_. On the other hand, when *θ*_*Y*_*i*__ = 0 the *i* ^*th*^ intervention reflects on *X*_*i*_ and on the previous updated times between interventions, composing a geometric propagation of the quality of the *i* ^*th*^ restoration on the overall system performance history. In general, *θ*_*Y*_*i*__ = 1 may characterize unintended (corrective) interventions where the causes of the system stoppage are investigated in order to restore its continuation condition, only. On the other hand, *θ*_*Y*_*i*__ = 0 must characterize intended (preventive) interventions where eventual previous negligence of the maintenance crew on parts of the system can be inspected, identified, and then suppressed. Thus, depending on *Y*_*j*_ (*e*.*g*. whether the *j* ^*th*^ intervention is planned or unplanned), the virtual age might be more or less affected by the *j* ^*th*^ intervention.

Still, [3] works with the GRP presenting a concept regarding virtual age functions considering the stochastic nature of the system restoration, but only considering the quality of the interventions according to *q*. This is the same kind of interpretation seen in [7], where models Kijima Type I and II are presented and their efficiency is separately studied. Another feature of traditional GRP modelling is that the virtual age of the system has been modelled by either Kijima Type I (*θ*_*Y*_*i*__ = 1) or Type II (*θ*_*Y*_*i*__ = 0) model. However, historical data might naturally involve a degrade between short (Type I) and long (Type II) memory-impact interventions. Thus, the traditional modelling does not allow the weight of both models in the analysis. Here, by means of Eq (1), we present a mixed version where such degrade is possible. In this way, the lesser *θ*_*Y*_*j*__ the greater the impact of the interventions of type *Y*_*j*_ on the system performance. Thus, the proposed model also allows one to compare the quality of the existing intervention types, aiming to supply some gaps in GRP modelling.

### The WGRP functions

The WGRP was firstly presented by [7] and then applied in some works as [8]. In WGRP, each time between interventions, *X*_*i*_, follows a Weibull distribution [15] conditioned on the corresponding virtual age, *v*_*i* − 1_, with shape and scale parameters *β* and *α*, in this order. Such a model is studied as follows.

We can define the Cumulative Distribution Function (CDF) for the WGRP as follows:

$$\begin{array}{cc} F_{T_{i}}\;(x+v_{i-1}|v_{i-1},\alpha ,\beta ) & =\frac{1-\text{exp}\;\left[-{\left(\frac{x+v_{i-1}}{\alpha }\right)}^{\beta }]-1+\text{exp}\;[-{\left(\frac{v_{i-1}}{\alpha }\right)}^{\beta }\right]}{1-1+\text{exp}\;\left[-{\left(\frac{v_{i-1}}{\alpha }\right)}^{\beta }\right]}\\ & =\frac{-\text{exp}\;\left[-{\left(\frac{x+v_{i-1}}{\alpha }\right)}^{\beta }]+\text{exp}\;[-{\left(\frac{v_{i-1}}{\alpha }\right)}^{\beta }\right]}{\text{exp}\;\left[-{\left(\frac{v_{i-1}}{\alpha }\right)}^{\beta }\right]}\\ & =1-\text{exp}\;\left[{\left(\frac{v_{i-1}}{\alpha }\right)}^{\beta }-{\left(\frac{x+v_{i-1}}{\alpha }\right)}^{\beta }\right] \end{array}$$ (2)

From the WGRP CDF, one can obtain the respective probability density function (PDF):

$$\begin{array}{c} f_{T_{i}}\;(x+v_{i-1}|v_{i-1},\alpha ,\beta )=\frac{\beta }{\alpha }{\;\left(\frac{x+v_{i-1}}{\alpha }\right)}^{\beta -1}\text{exp}\;\left[{\left(\frac{v_{i-1}}{\alpha }\right)}^{\beta }-{\left(\frac{x+v_{i-1}}{\alpha }\right)}^{\beta }\right] \end{array}$$ (3)

In turn, in the light of *F*_*T*_*i*__(*x*+*v*_*i*−1_ ∣ *v*_*i*−1_, *α*, *β*) and *f*_*T*_*i*__(*x*+*v*_*i*−1_ ∣ *v*_*i*−1_, *α*, *β*), it is straightforward to obtain the respective hazard function:

$$\begin{array}{c} h_{T_{i}}\;(x+v_{i-1}|v_{i-1},\alpha ,\beta )=\frac{f_{T_{1}}\;(x+v_{i-1}|v_{i-1},\alpha ,\beta )}{1-F_{T_{1}}\;(x+v_{i-1}|v_{i-1},\alpha ,\beta )}=\frac{\beta }{\alpha }{\;(\frac{x+v_{i-1}}{\alpha })}^{\beta -1} \end{array}$$ (4)

From Eq (4) it is possible to analyze the meaning of the parameters according to the behaviour of the system in response to the interventions. Clearly, there are three situations: when *β* > 1, *β* = 1, and *β* < 1. If *β* < 1, the greater the time (actual plus virtual ones) the lesser the hazard; thus the greater the *q* the better the interventions. On the other hand, if *β* > 1, the greater the *q* (and thus the time) the greater the hazard. Finally, if *β* = 1 the system is stable and therefore has no memory. Thus, the values of *β* and *q* might reflect the relationship between the system and the interventions. In fact, this reasoning brings a more general interpretation for the meaning of the WGRP parameters in relation to the WGRP literature, mostly dedicated to the cases where *β* > 1.

**Fig 1:**
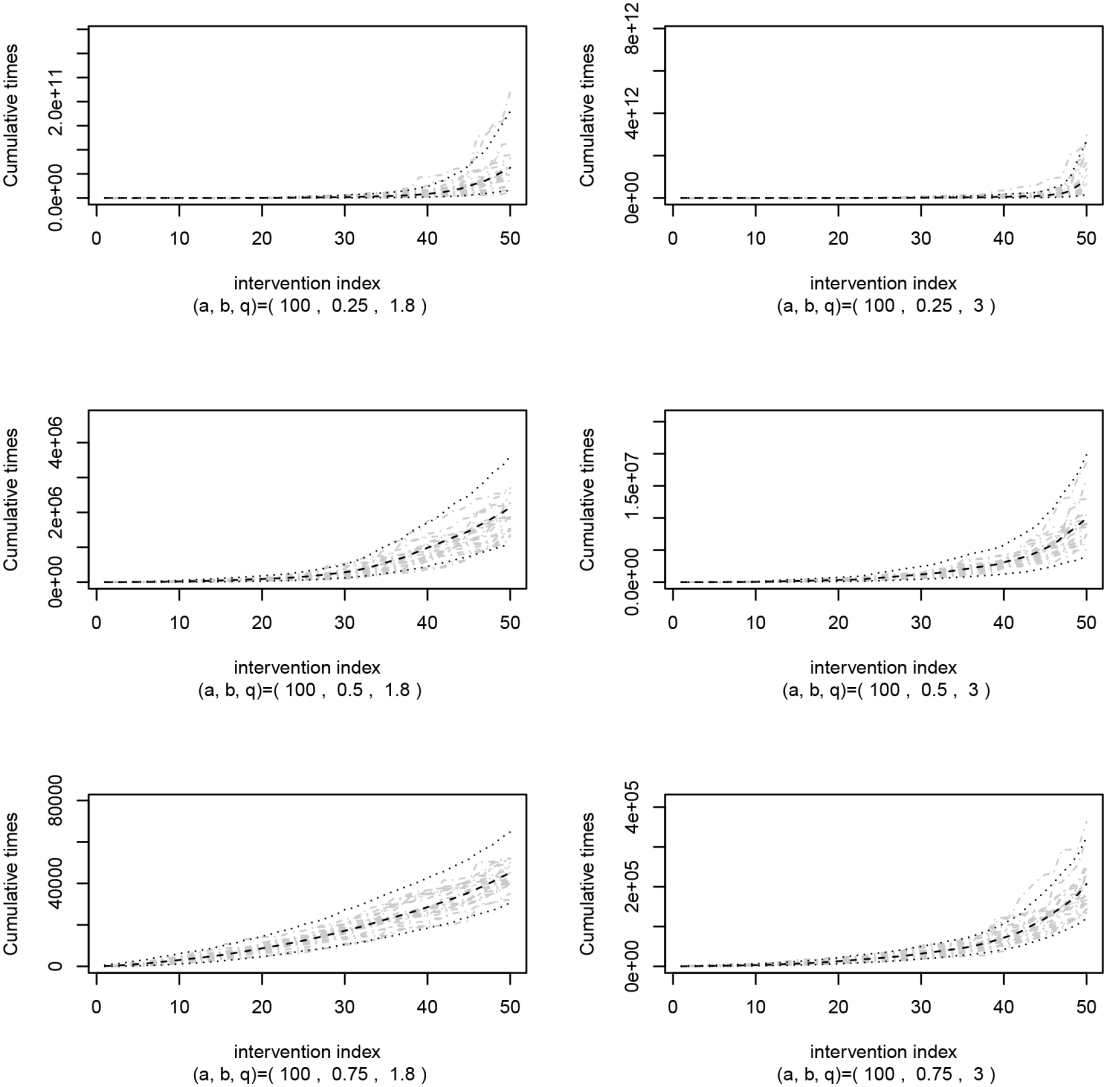
Explaining relationship. WGRP samples to explain relationship between values of *β* < 1 and *q* > 1.

To reinforce these perspectives, Fig 1 presents some simulations involving the relationship between *β*, *q*, and the stoppage times. In fact, as previously stated, if *β* < 1 then the greater the *q* the greater the level of system improvement imposed by the interventions, once times between interventions enlarge. Thus, it is claimed that the literature traditionally interpret *q* for *β* > 1 only, and neglects the meaning of *q* in the cases where *β* < 1.

Thus, it is more reasonable to think of *β* as being the main parameter for reflecting the system restoration pattern instead of *q*. In this way, the meaning of *q* depends on *β*. For instance, when *β* = 1, *q* becomes useless and the times between interventions are identically and exponentially distributed. In this situation the system is considered memoryless once the hazard function is constant through the time (it only depends on *α*). Obviously, this case brings important discussions. In particular, it must reflect the situation where the intrinsic nature of the system as well as the performance of its maintenance crew are aligned in such a way that the system stays stable over the time. It would be seen as some kind of perfect balance, something possible though rare. These discussions make arise the importance of a correct interpretation of the WGRP parameters, mainly *q*. In fact, *α* and *β* can be understood similarly to the case of the Weibull distribution.

On the other hand, the mathematical challenges of WGRP has not been fully addressed. [10] present an asymptomatic claim about the complexity of working mathematically with GRP. These authors state that the closed-form solution of the mean time between interventions is not available. In fact, there is little or even no work in WGRP literature that develops math properties like the first moments. This problem is handled as follows.

### First and second theoretical moments of WGRP

These moments might be important to promote preventive interventions analyzes. In this way and without loss of generality the index of *v*_*i*−1_ is suppressed. The first moment is developed as follows:

$$\begin{array}{ll} 𝔼(X|\alpha ,\beta ,v) & =\int_{0}^{\infty }x\frac{\beta }{\alpha }{\left(\frac{x+v}{\alpha }\right)}^{\beta -1}\text{exp}\;[{\left(\frac{v}{\alpha }\right)}^{\beta }-{\left(\frac{x+v}{\alpha }\right)}^{\beta }]\;dx\\ & =\text{exp}\;\left[{\left(\frac{v}{\alpha }\right)}^{\beta }\right]\;\int_{0}^{\infty }x\frac{\beta }{\alpha }{\left(\frac{x+v}{\alpha }\right)}^{\beta -1}\text{exp}\;\left[-{\left(\frac{x+v}{\alpha }\right)}^{\beta }\right]\;dx\\ & =\text{exp}\;\left[{\left(\frac{v}{\alpha }\right)}^{\beta }\right]\{\int_{-v}^{\infty }x\frac{\beta }{\alpha }{\left(\frac{x+v}{\alpha }\right)}^{\beta -1}\text{exp}\;\left[-{\left(\frac{x+v}{\alpha }\right)}^{\beta }\right]\;dx\\ & \;-\int_{-v}^{0}x\frac{\beta }{\alpha }{\left(\frac{x+v}{\alpha }\right)}^{\beta -1}\text{exp}\;\left[-{\left(\frac{x+v}{\alpha }\right)}^{\beta }\right]\;dx\} \end{array}$$

Using some algebra and substitution, one has:

$$𝔼(X|\alpha ,\beta ,v)=\text{exp}\;\left[{\left(\frac{v}{\alpha }\right)}^{\beta }\right]\;\left\{\alpha \Gamma \;\left(1+\frac{1}{\beta }\right)+\frac{\alpha }{\beta }\tilde{\Gamma }\;\left(\frac{1}{\beta },{\;\left(\frac{v}{\alpha }\right)}^{\beta }\right)-\frac{\alpha }{\beta }\tilde{\Gamma }\left(\frac{1}{\beta },0\right)\right\}$$ (5)

and 𝔼(*X*+*v* ∣ *α*, *β*, *v*) = 𝔼(*X* ∣ *α*, *β*, *v*)+*v*.

In turn, the non-central second moment is given by:

$$\begin{array}{ll} 𝔼({\left(X+v\right)}^{2}|\alpha ,\beta ,v) & =\int_{0}^{\infty }{\left(x+v\right)}^{2}f(x+v|\alpha ,\beta ,v)dx\\ & =\int_{0}^{\infty }x^{2}f(x+v|\alpha ,\beta ,v)dx+2v\int_{0}^{\infty }xf(x+v|\alpha ,\beta ,v)dx\\ & \;+v^{2}\int_{0}^{\infty }f(x+v|\alpha ,\beta ,v)dx\\ & =\text{exp}\;[{\left(\frac{v}{\alpha }\right)}^{\beta }]\left\{\int_{-v}^{\infty }x^{2}\tilde{f}(x|\alpha ,\beta ,v)dx-\int_{-v}^{\infty }x^{2}\tilde{f}(x|\alpha ,\beta ,v)dx\right\}\\ & \;+2v𝔼(x+v|\alpha ,\beta ,v)+v^{2}. \end{array}$$

Finally, one has:

$$\begin{array}{l} 𝔼\left({\left(X+v\right)}^{2}\right)=\text{exp}\;\left[{\left(\frac{v}{\alpha }\right)}^{\beta }\right]\;\{\alpha^{2}\Gamma \left(1+\frac{2}{\beta }\right)+\\ \;+v^{2}+2\overset{0}{\underset{-v}{\int }}x\;\text{exp}\;[{\left(\frac{x+v}{\alpha }\right)}^{\beta }]\;dx\}+2v\cdot 𝔼\left(X\right)+v^{2}. \end{array}$$ (6)

These calculations conclude the first and second moments of *X*. As can be seen, these moments involve the exponential and the incomplete gamma functions. The presence of *v* in these functions may lead to computational problems as *v* increases. Further, the incomplete Gamma function is given by $\tilde{\Gamma }(a,z)=\int_{a}^{\infty }e^{-t}t^{z-1}dt$ and must be approximated via numeric calculus.

Next section discusses some numerical results where the maximum likelihood estimation (MLE) and the random sampling problems are studied.

### MLE process for the WGRP parameters set

The estimation process is presented to illustrate how the parameters are obtained through the joint PDF and derivatives of WGRP, via MLE:

$$f\left(x\;|\;\alpha ,\beta ,q\right)=\frac{\beta^{n}}{\alpha^{n\beta }}\;[\prod_{i=1}^{n}{\left(x_{i}+v_{i-1}\right)}^{\beta -1}]\;e^{\frac{1}{\alpha^{\beta }}\;(\sum_{i=1}^{n}v_{i-1}^{\beta }-\sum_{i=1}^{n}{\left(x_{i}+v_{i}-1\right)}^{\beta })}$$ (7)

It is worthwhile to mention that *v*_*i*_ encapsulates the maintenances history of the system until *i* ^*th*^ intervention. Let ℓ = ln *f* denote the log-likelihood function underlying the WGRP. Then:

$$\ell =n\left(\ln\beta -\beta \ln\alpha \right)+\left(\beta -1\right)\sum_{i=1}^{n}\ln\left(x_{i}+v_{i-1}\right)+\frac{1}{\alpha^{\beta }}\;[\sum_{i=1}^{n}v_{i-1}^{\beta }-\sum_{i=1}^{n}{\left(x_{i}+v_{i-1}\right)}^{\beta }]$$ (8)

From ℓ, one can obtain the MLE for *α* by computing the *α* for which $\frac{d\ell }{d\alpha }=0$:

$$\hat{\alpha }={\left[\frac{\sum_{i=1}^{n}{\left(x_{i}+{\hat{v}}_{i-1}\right)}^{\hat{\beta }}-\sum_{i=1}^{n}{\hat{v}}_{i-1}^{\hat{\beta }}}{n}\right]}^{\frac{1}{\hat{\beta }}}.$$ (9)

From Eqs (7), (8), and (9) it is shown the MLE process to infer *α*. However, the MLE process to estimate *β* and *q* is mathematically intriguing. Further, it can be seen from Eqs (10) and (11) the reference to the derivatives of Eq (8) to obtain the MLE for *β* and *q*. Only the derivatives are presented here once it is not possible to analytically isolate *β* and *q* as done with *α*. Thus, for practical purposes, $\hat{\alpha }$, $\hat{\beta }$, and $\hat{q}$ can be approximated via probabilistic optimization algorithms (*e*.*g*. simulated annealing, particle swarm, and genetic algorithms), where $\hat{\alpha }$ is a deterministic function of $\hat{\beta }$ and $\hat{q}$ via Eq (9).

$$\begin{array}{ll} \frac{d\ell }{d\beta } & =\frac{n}{\hat{\beta }}-n\text{ln}\hat{\alpha }+\sum_{i=1}^{n}\text{ln}(x_{i}+{\hat{v}}_{i-1})\\ & \;-{\hat{\alpha }}^{-\hat{\beta }}\text{ln}\hat{\alpha }\;[\sum_{i=1}^{n}{\hat{v}}_{i-1}^{\hat{\beta }}-\sum_{i=1}^{n}{\left(x_{i}+{\hat{v}}_{i-1}\right)}^{\hat{\beta }}]\\ & \;+\frac{1}{{\hat{\alpha }}^{\hat{\beta }}}\;[\sum_{i=1}^{n}{\hat{v}}_{i-1}^{\hat{\beta }}\text{ln}\sum_{i=1}^{n}{\hat{v}}_{i-1}-\sum_{i=1}^{n}{\left(x_{i}+{\hat{v}}_{i-1}\right)}^{\hat{\beta }}\text{ln}\sum_{i=1}^{n}\left(x_{i}+{\hat{v}}_{i-1}\right)] \end{array}$$ (10)

and

$$\frac{d\ell }{dq}=\left(\hat{\beta }-1\right)\sum_{i=1}^{n}\frac{{\hat{v}}_{i-1}^{\prime }}{x_{i}+{\hat{v}}_{i-1}}+\frac{1}{{\hat{\alpha }}^{\hat{\beta }}}\;[\sum_{i=1}^{n}\hat{\beta }{\hat{v}}_{i-1}^{\hat{\beta }-1}{\hat{v}}_{i-1}^{\prime }-\sum_{i=1}^{n}\hat{\beta }{\left(x_{i}+{\hat{v}}_{i-1}\right)}^{\hat{\beta }-1}{\hat{v}}_{i-1}^{\prime }]$$ (11)

Despite the parameter *q* is not explicitly showed in the derivatives, it is intuitive to notice that $\hat{v}$ encapsulates it by means of the mixed model in Eq (1).

The parameter *θ*_*y*_*i*__ presented in the mixed virtual age (in Eq (1)) is also estimated through probabilistic optimization and it belongs to the range [0, 1] for each evidenced intervention type *y*_*i*_. Thus, for *y*_*i*_, the value of *θy*_*i*_ is optimized aiming to adjust the proportion of each Kijima model present on that intervention type.

### WGRP random sampling process

Sampling random WGRP series is made by means of the inverse transform method [16]. Specifically, this method is based on the equality *u* = *R*(*x*+*v* ∣ *v*), where *R*(*x*+*v* ∣ *v*) = 1 − *F*(*x*+*v* ∣ *v*) denotes the WGRP reliability(survival) function. In the inverse transform method one has that *u* is an instance of the random variable *U* ∼ *Uniform* [0, 1]. So, by isolating *x* (resorting to Eq (2) one has the WGRP instance

$$x=\alpha {\;[{\left(\frac{v}{\alpha }\right)}^{\beta }-\text{ln}\left(u\right)]}^{\frac{1}{\beta }}-v.$$ (12)

Therefore, it is allowed through Eq (12) one to generate a sequence of *n* times between interventions, (*x*_1_, ⋯, *x*_*i*_, ⋯, *x*_*n*_), according to instances of *α*, *β*, *q*, (*y*_1_,⋯, *y*_*i*_,⋯, *y*_*n*_), and (*θ*_*y*_1__,⋯, *θ*_*y*_*i*__,⋯, *θ*_*y*_*n*__). By generating such sequence of WGRP random numbers, it is possible to notice the implications of the inverse *Gamma* function. Here, this sequence is such useful to show the behaviour of the theoretical first moment developed in previous section. Furthermore, one can notice an important behaviour of the system according with the values of *q* and *β* in WGRP. Theoretically, from Eq (4), one can see that depending on the value of *β*, the WGRP hazard function presents an increasing (for *β* > 1) or decreasing (*β* < 1) behaviour, since the remaining arguments of *h*_*T*_*i*__(⋅) are non-negative. In this way, once the virtual age is proportional to *q* in Kijima models (Eq (1), if *β* < 1 the greater the value of *q*, the greater the level of improvement of the system. Such a reasoning seems to be wrongly neglected by WGRP practitioners. In fact, it has been usual to interpret increased values of *q* as a low performance metric for the maintenance crew, though its meaning is strongly dependent on the value of *β*. From Eq (4) one has sufficient arguments for reinterpreting the meaning of *q*, depending on *β*. Thus, generating time between interventions can demonstrate numerically this reasoning.

**Fig 2:**
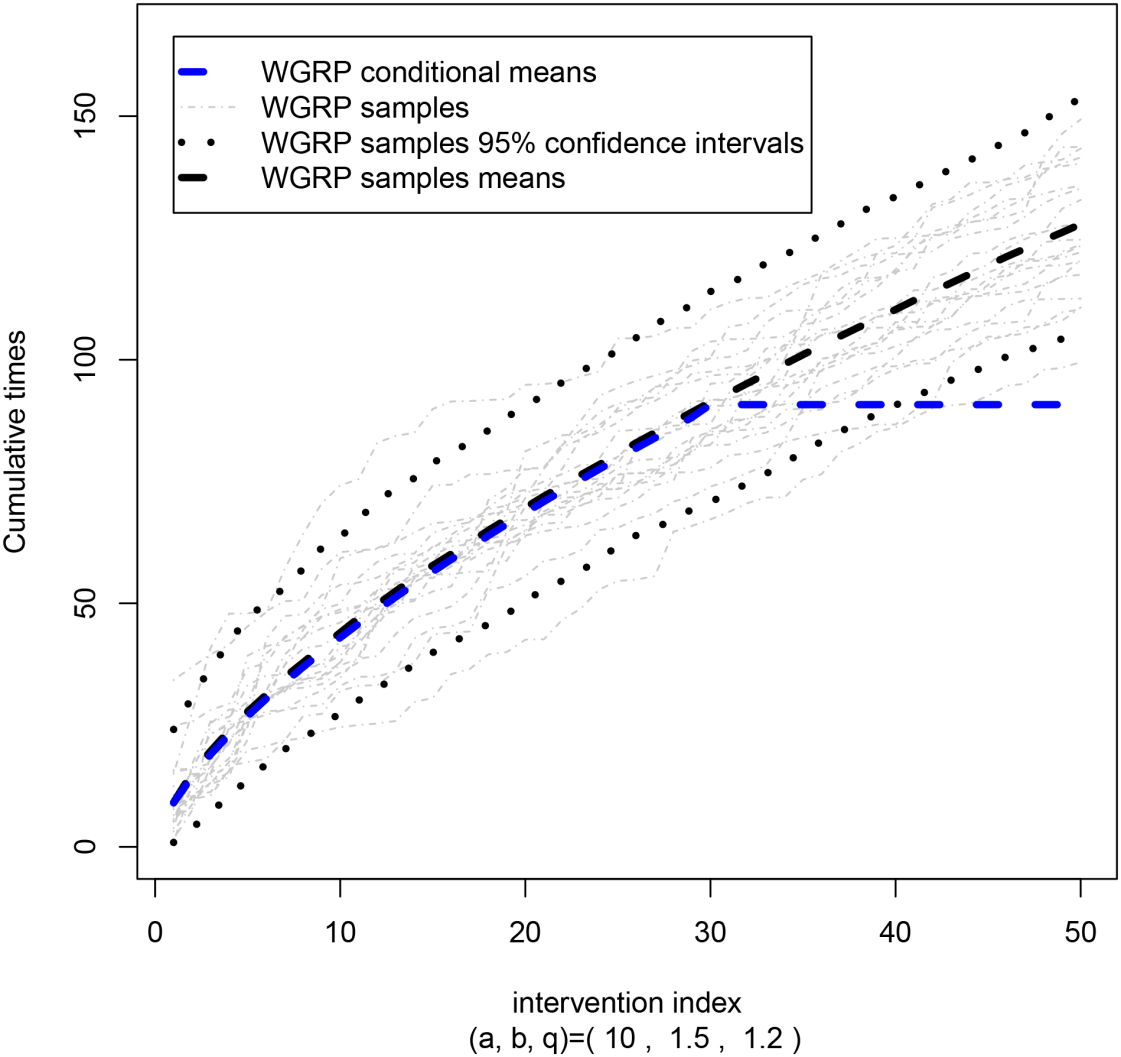
Sampling and Conditional Moments. WGRP samples statistics and theoretical conditional expected values in an aging system (*β* > 1).

Considering an aging system where *β* > 1, *q* = 1.2, and *θ*_*y*_*i*__ = 1 (Fig 2), the times between interventions becomes closer gradually and the impact of the interventions is not so worth (*q* = 1.2). In other words, next intervention must be made earlier than the previous one and the intervention actions have contributed to such behaviour (otherwise, we wold have *q* near 0). Furthermore, since *β* > 1, the power term in the first moment grows rapidly and the theoretical expected value quickly becomes intractable due to the complex math terms (see blue line).

## Real world cases

In this section some results involving the adjustment of a WGRP model to three real cases are presented. Specifically, these cases present an increasing, constant and a decreasing hazard function. The RP, NHPP, Kijima I, Kijima II, and proposed models were adjusted for each data set, via MLE, where the log-likelihood function, Eq (8), was optimized according to the simulated annealing algorithm provided by the GenSA package of the free-ware R software [17].

For the sake of comparison, the mean squared error (MSE) and log-likelihood (LL) metrics were computed for each model. Regarding the MSE, it was based on mean stoppage times, estimated from 200,000 simulated samples from each model. In this context, it was considered the following space of possibilities for $\hat{\beta }$, $\hat{q}$, and $\hat{\theta }$: $(\hat{\beta },\hat{q},\hat{\theta })\in [10^{-100},10]\times [-1.5,1.5]\times {\left[0,1\right]}^{k}$, where *k* is the number of alternatives for intervention.

### Analyses

#### offshore facility data set

The first data set is from [18] where 84 stoppage times, regarding two intervention types (corrective(*c*) and preventive (*p*)) of a compressor system of an offshore facility is considered. Thus, the data set records two variables: times between maintenance actions and the respective types of maintenance. It is worthwhile to mention that the time until first intervention (*t*_1_ = 220) was removed from the modelling study once it was considered an outlier. Thus, *X*_0_ = *t*_1_ in this case.

Firstly, the RP, NHPP, Kijima I, Kijima II, and proposed models were adjusted to the data set. Further, the performance measures (LL and MSE) of each model were computed (see Table 1), via MLE. It is possible to conclude that the Kijima II model (the proposed one where *θ* = (0,0)) presents the best performance in terms of both LL and MSE.

**Table 1:** MLE parameters estimates, MSE and Log-Likelihood (LL) measures of WGRP models for the offshore facility dataset from [18].

Model	$\hat{\alpha }$	$\hat{\beta }$	$\hat{q}$	${\hat{\theta }}_{c}$	${\hat{\theta }}_{p}$	MSE	LL
RP	14.39	0.79	*	*	*	49185.97	-312.27
NHPP	2.39	0.69	*	*	*	13991.01	-310.64
Kijima I	3.299	0.52	0.02	1	1	18542.23	-306.74
Kijima II	5.85	0.95	1.499	0	0	**	**
Mixed Kijima Model	5.85	0.95	1.499	0	0	3346.835	-306.63

Estimated parameters, MSE and LL measures for Oil offshore dataset in [18].

* reflects the absence of this value in the model.

** means the same value found for both Kijima II and Mixed Kijima model.

**Fig 3:**
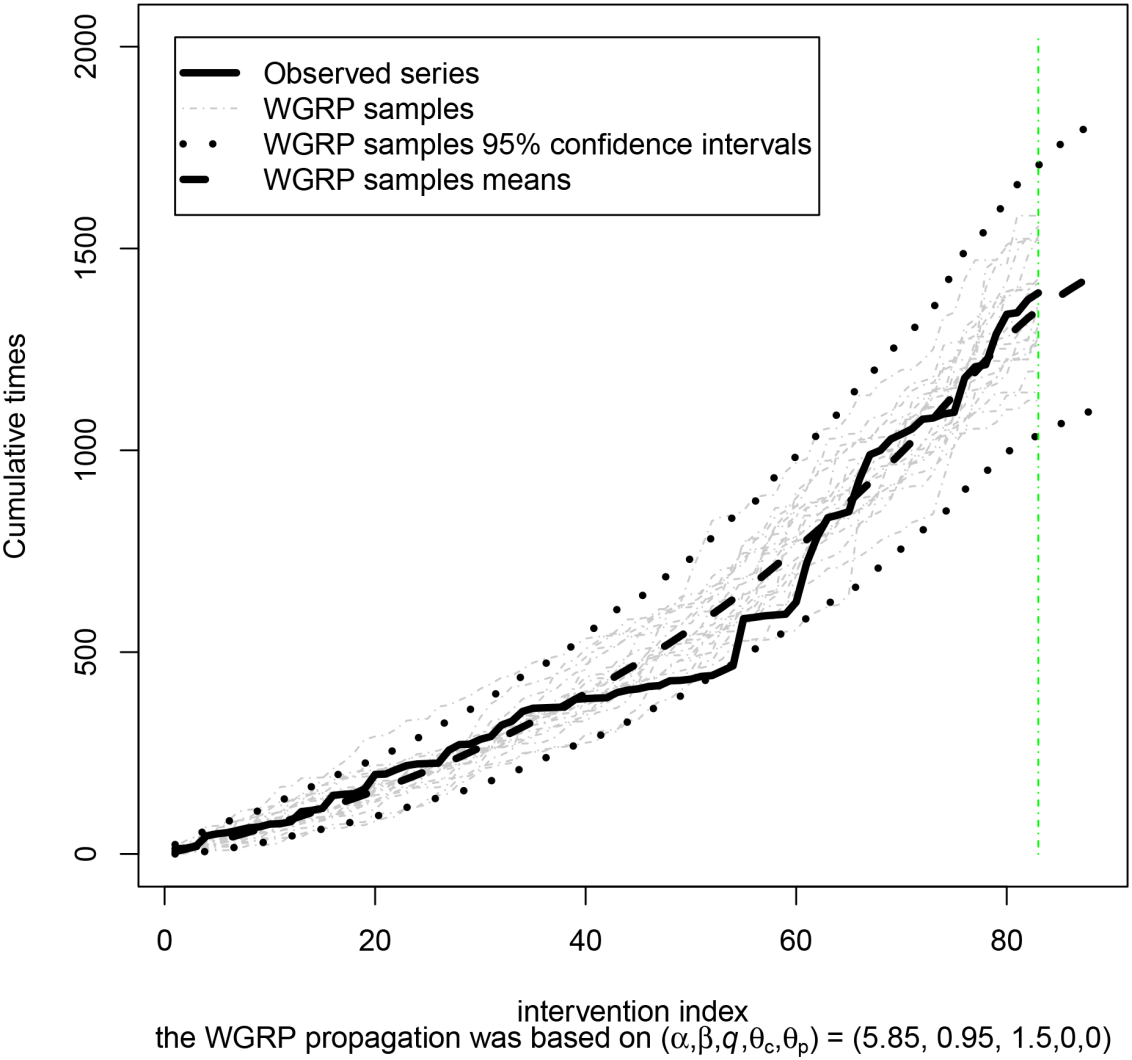
WGRP adjustment for Offshore. WGRP adjustment to the [18] Offshore oil data set without *t*_1_.

Considering the proposed model, the maximum likelihood estimates for *α*, *β*, *q*, and *θ* = (*θ*_*c*_, *θ*_*p*_) from the adopted data set (without *t*_1_) were $\left(\hat{\alpha },\hat{\beta },\hat{q},({\hat{\theta }}_{c},{\hat{\theta }}_{p}))=(5.87,0.95,1.5,(0.0,0.0)\right)$. Thus, due to *θ* estimates, the proposed approach has been simplified to the Kijima II model. As $\hat{\beta }<1.0$, it follows that the system is improving, in the sense that the greater the time the lower the hazard rate. It might reflect a burn-in period of the system, characterized by early failures attributable to defects in design, manufacturing, or construction [19]. In turn, (*q*, *θ*_*c*_, *θ*_*p*_) = (1.5,0.0,0.0) reveals that regardless the intervention type (whether corrective or preventive), its positive impact propagates through the entire system history with the maximum intensity, once *v*_*i*_ = 1.5 ⋅ (*v*_*i* − 1_+*x*_*i*_) and it was assumed *q* ≤ 1.5. It is scratched in Fig 3 the observed stoppage time series and instances of the best fitted model.

In fact, it is exhibited in Fig 3 the observed cumulative times ***t*** = (*t*_2_, *t*_3_, …, *t*_85_), the respective WGRP sample points and 95% interval estimates as well as some of the series simulated from the fitted model. In this case, precise estimates from Eq (5) were unavailable due to computational limitations. One can see that the fitted model has enveloped the performance time series data set in such a way that the series is always in its 95% confidence interval estimates and the generated samples are similar to the real series, which indicates that the proposed WGRP model with $\left(\hat{\alpha },\hat{\beta },\hat{q},({\hat{\theta }}_{c},{\hat{\theta }}_{p}))=(5.87,0.95,1.5,(0.0,0.0)\right)$ appears to be a suitable model for this situation.

Besides, it is also sketched in Fig 3 the proposed preventive maintenances policy for the next 4 interventions, according to the adjusted model. Although ***t*** does not involve *t*_1_, to sum up *t*_1_ to the proposed preventive interventions policy, from the 85^*th*^ to the 88^*th*^ one, is a straightforward way for circumventing the problem. It is presented in Table 2 the proposed instants for preventive interventions. Thus, in average, it is suggested, for instance, that the next preventive intervention should be performed in the instant 1657.7; however, it is inferred the system becomes unavailable at any instant in the interval [1343.9,2016.25], under a 95% confidence level. This provides some important conclusions, since the decision making can be based on these estimates, leading the maintenance crew to be under alert mode during these time interval.

**Table 2:** Policy for the next four preventive interventions of the system studied by [18].

intervention	85	86	87	88
2.5% quantile	1343.9	1399.76	1445.47	1513.81
mean	1657.7	1721.86	1801.9	1909.78
97.5% quantile	2016.25	2114.38	2261.27	2436.53

#### Windshield data set

The second data set involves 80 stoppage times regarding failure (say *f*) and service (say *s*) actions on a windshield system, from [20]. Table 3 brings the MLE estimates of the alternative models and their respective log-likelihood and MSE metrics. The maximum likelihood estimates of *α*, *β*, *q* and (*θ*_*f*_, *θ*_*s*_) to the proposed model are $\left(\hat{\alpha },\hat{\beta },\hat{q},({\hat{\theta }}_{f},{\hat{\theta }}_{s})\right)$ = (0.06, 1.04, 1.5, (0.0,0.0). Similarly to the previous case, the proposed model has been specified to the Kijima II approach and achieved the best results in terms of both LL maximization and MSE minimization.

**Table 3:** MLE parameters estimates of the WGRP models and respective MSE and LL measures for the Windshield data set from [20].

Model	$\hat{\alpha }$	$\hat{\beta }$	$\hat{q}$	${\hat{\theta }}_{f}$	${\hat{\theta }}_{s}$	MSE	LL
RP	0.028	0.897	*	*	*	0.115	201.93
NHPP	0.119	1.465	*	*	*	0.0158	206.19
Kijima I	0.11	1.489	0.66	1	1	0.0151	206.22
Kijima II	0.0598	1.044	1.495	0	0	**	**
Mixed Kijima Model	0.0598	1.044	1.495	0	0	0.0053	207.99

Estimated parameters, MSE and LL measures for Windshield dataset.

* reflects the absence of this value in estimation process.

** means the same value found for both Kijima II and Mixed Kijima model.

As $\hat{\beta }>1$, it is inferred the system is deteriorating; thus the greater the time the greater the hazard. It might represent a wear-out phase, mainly characterized by complex aging phenomena, where the system deteriorates (e.g., due to accumulated fatigue) and is more vulnerable to outside shocks [19]. In turn, $\left(\hat{q},{\hat{\theta }}_{f},{\hat{\theta }}_{s})=(1.5,0.0,0.0\right)$ indicates that regardless the intervention type (whether due to failure or service), its negative impact propagates through the entire system history with the maximum possible intensity, oppositely to the offshore facility case. This is caused due to the increasing hazard function ($\hat{\beta }>1$), the deteriorating interventions ($\hat{q}>1$), and the adoption of the Kijima II model ($\hat{\theta }=(0.0,0.0)$). Thus, it is advised here the study of different ways to intervene, once the current ones seem to contribute to the system deterioration.

**Fig 4:**
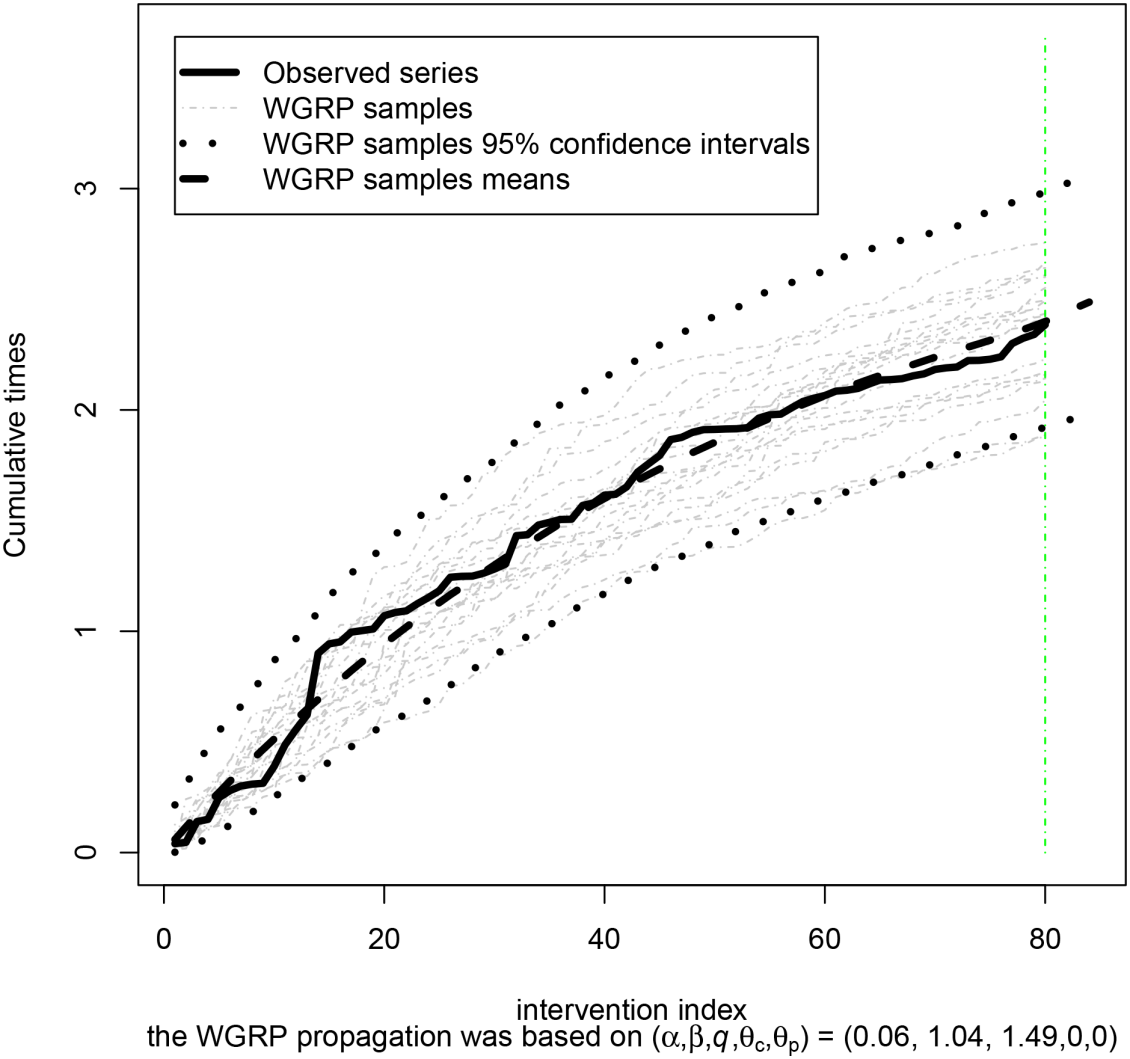
WGRP adjustment for Windshield. WGRP adjustment to the [20] Windshield data.

It is illustrated in Fig 4 the observed cumulative times ***t*** = (*t*_2_, *t*_3_, …, *t*_80_), the respective WGRP sample points and 95% interval estimates as well as some of the series simulated from the fitted model. Further, it is presented in Table 4 4 forecasts from this model for the next times between interventions.

**Table 4:** Preventive interventions policy for the windshield data set from [20] according the best WGRP model.

intervention	81	82	83	84
2.5% quantile	1.93	1.95	1.97	2.01
mean	2.41	2.44	2.47	2.51
97.5% quantile	3	3.02	3.05	3.12

Policy for the next four preventive interventions of the system studied by Windshield data.

#### Transformers data set

This data set corresponds to 61 transformers stoppage events presented by [21], the least data set considered in the paper. Here, the intervention type is related to the complexity of the respective system: whether monophasic (the simplest case, represented here by letter *m*), or three-phase (say *t*). In Table 5, one can see the MLE parameters estimates for each model and the respective performance metrics. The proposed model presented the maximum LL and minimum MSE.

**Table 5:** MLE estimates, MSE and LL measures of the WGRP models with respect to the Transformers dataset from [21].

Model	$\hat{\alpha }$	$\hat{\beta }$	$\hat{q}$	${\hat{\theta }}_{m}$	${\hat{\theta }}_{t}$	MSE	LL
RP	179.77	1.588	*	*	*	52929.59	-363.43
NHPP	227.14	1.088	*	*	*	23521.41	-371.26
Kijima I	210.34	1.91	0.005	1	1	21664.52	-361.78
Kijima II	273.11	2.336	0.3805	0	0	22094.55	-361.58
Mixed Kijima Model	282.53	2.519	0.2378	0.449	0.589	21436.53	-360.59

Estimated parameters, MSE and LL measures for Transformers dataset.

* means the absence of this value in estimation process.

Thus, differently from the previous cases, now the proposed approach suggests a degrade between the Kijima models for each type of intervention. The maximum likelihood estimates of *α*, *β*, *q*, and (*θ*_*m*_, *θ*_*t*_) are $\left(\hat{\alpha },\hat{\beta },\hat{q},({\hat{\theta }}_{m},{\hat{\theta }}_{t})\right)$ = (282.53, 2.52, 0.24, (0.45, 0.59)). As $\hat{\beta }>1$, we conclude that the system is deteriorating. Thus, the longer the time, the greater the hazard. However, as $\hat{q}\in (0.0,1.0)$, the interventions have restored the system to an intermediate condition, between “as good as new”(where *q* = 0) and “as bad as old”(where *q* = 1).

In turn, as ${\hat{\theta }}_{m}<{\hat{\theta }}_{t}$ and the lesser the *θ* the greater the positive impact of the intervention (approaching a Kijima II model), one can conclude that interventions on the monophasic items (relative to *θ*_*m*_) promote more restoration on the transformers system than the interventions on the three-phase ones (relative to *θ*_*t*_). Such a phenomenon might result from the different levels of difficult in performing interventions on monophasic and three-phase systems as well as from the skill of the maintenance team for dealing with these scenarios. Thus, the proposed model also allows to measure and compare the quality of the different types of intervention, promoting support to decide about crew training and evaluation.

**Fig 5:**
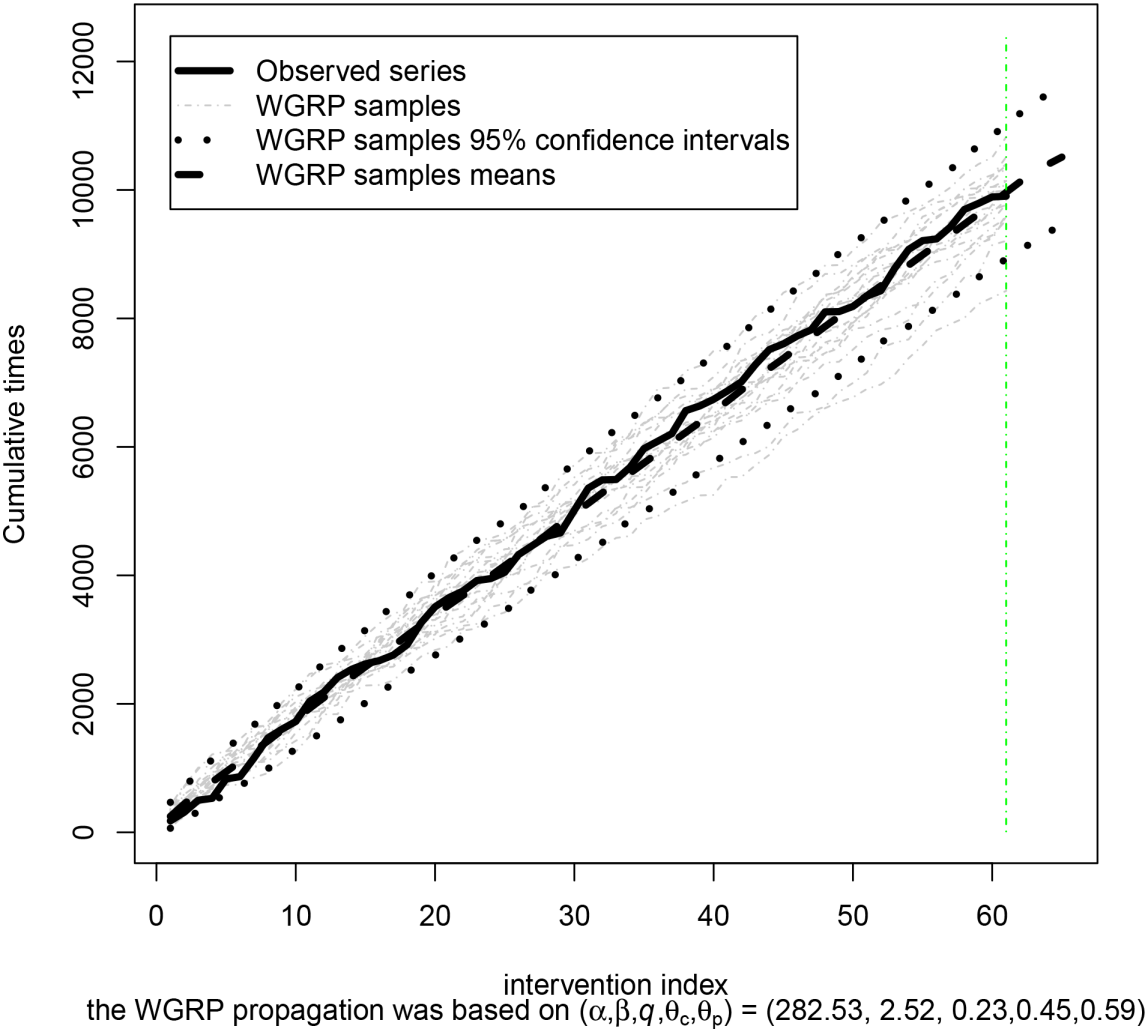
WGRP adjustment for Transformers. WGRP adjustment to the [21] Transformers data.

In Fig 5, it is exhibited the observed cumulative times ***t*** = (*t*_2_, *t*_3_, …, *t*_61_), the respective WGRP sample points and 95% interval estimates as well as some of the series simulated from the fitted model. From Fig 5, one could infer that RP is adequate to the performance data set. However, it is clear from Table 5 that the RP is not among the best models. In fact, RP is the worst model in terms of MSE and the second worst with respect to LL. It allows one to conclude, from the best models, that there is a trade-off between the maintenance interventions and the deterioration underlying the system, leading to an apparent phase of constant-value hazard function. Such cases are characterized by random failures of the component; in this period, many mechanisms of failure due to complex underlying physical, chemical, or nuclear phenomena give rise to this approximately constant-value hazard function [19]. 4 forecasts from this model for the next times between interventions are presented in Table 6.

**Table 6:** Preventive interventions policy for the Transformers data from [21], according to the best WGRP model.

intervention	62	63	64	65
2.5% quantile	9064.05	9200.17	9336.77	9456.1
mean	10126.57	10273.83	10397.75	10510.76
97.5% quantile	11192.19	11334.12	11504.78	11602.52

Policy for the next four preventive interventions of the system studied by Transformers dataset.

## Discussions and Conclusions

This paper presents new results for GRP modelling in both theoretical and numerical perspectives. Theoretically, the paper overcomes the challenge of deciding between Kijima types I and II models when fitting the impact of the interventions through the system history, by introducing a mixed virtual age model. Thus, a set of new parameters, say *θ* = (*θ*_1_,⋯, *θ*_*k*_), corresponding to the weight of the Kijima models for each one of the *k* existing intervention types, is introduced.

Further, the paper emphasizes the interpretation of the resulting WGRP parameters (*e*.*g*. *θ* and *q*, depending on *β*) and unprecedentedly introduces the math developments for the first and the second moments of the WGRP, showing its computational limitations. Besides, some numerical calculations are presented to demonstrate how this moments behave along the time and showing an inverse method to generate random values from the proposed mixed model.

Finally, the usefulness of WGRP was illustrated by modelling three real world cases from literature. In this way, stable, improving and deteriorating systems were studied as well as the contributions of the intervention actions to the systems performance. Due to the presence of *θ* in the mixed WGRP model, it was possible to distinguish the quality of each intervention type by capturing the proportional adjustment of each Kijima model. Specifically, we verified an improving offshore compressor system involving adequate corrective and maintenance interventions; an aging windshield system involving inadequate failure and service actions; and an deteriorating though stable electric system where the intervention quality depends on the nature of the demanding subsystem.

Furthermore, a preventive interventions policy was elaborated to each system, in order to illustrate the forecasting usefulness of the proposed model.

Ongoing researches address the introduction of the proposed mixed model in the competitive risks framework, since it can work with different kinds of interventions. Alternative virtual age functions and the Gamma distribution-based GRP are also in development by the authors.
